# Discovery of protease inhibitors from bacteria as novel adjuvants for oral vaccine formulations

**DOI:** 10.3389/fimmu.2025.1679540

**Published:** 2025-10-21

**Authors:** Lorena M. Coria, Celeste Pueblas Castro, Laura Bruno, Helder I. Nakaya, Karina A. Pasquevich, Juliana Cassataro

**Affiliations:** ^1^ Instituto de Investigaciones Biotecnológicas, Universidad Nacional de San Martín (UNSAM) – Consejo Nacional de Investigaciones Científicas y Técnicas (CONICET), Buenos Aires, Argentina; ^2^ Escuela de Bio y Nanotecnología (EByN), Universidad Nacional de San Martín, Buenos Aires, Argentina; ^3^ Hospital Israelita Albert Einstein, São Paulo, Brazil; ^4^ Institut Pasteur de São Paulo, São Paulo, Brazil; ^5^ Department of Clinical and Toxicological Analyses, School of Pharmaceutical Sciences, University of São Paulo, São Paulo, Brazil

**Keywords:** protease inhibitor, mucosal adjuvant, oral delivery, antigen degradation, mucosal immunity

## Abstract

**Introduction:**

There is a need for mucosal vaccines that can fight pathogens at the site of infection. At present, there are no approved adjuvants for mucosal vaccines. Among different immunization routes, oral delivery is the natural choice because of its ease of administration. However, oral administration has two main drawbacks: proteolytic digestion and immune tolerance.

**Methods:**

In this study, a systematic *in silico* screening of putative protease inhibitors (PIs) from bacteria to identify novel oral vaccine adjuvants was conducted. Selected candidates were then evaluated for their ability to inhibit gastrointestinal proteases and to stimulate murine dendritic cells. Finally, promising candidates were incorporated as adjuvants into oral vaccine formulations containing model (OVA) or real antigens, such as the cholera toxin B subunit (CTB) and tetanus toxoid and tested in *in vivo* experiments. In addition, a proteomic analysis to assess their effects on dendritic cells was performed.

**Results:**

This approach led to the selection of 11 PIs from human pathogenic bacteria, representing diverse families of PIs. These proteins were then expressed in E. coli; five of them demonstrated soluble expression and efficient purification. Three candidates -Ecotin from Salmonella, APRin from Pseudomonas, and STA (staphostatin A) from Staphylococcus aureus- exhibited both protease inhibition and TLR4-independent dendritic-cell activation. *In vivo* studies demonstrated that Ecotin, APRin, and STA enhanced immune responses when orally co-administered with OVA, promoting T-cell proliferation and antibody production. Further evaluation with real antigens, confirmed their adjuvant effect by inducing mucosal and systemic immunity. Proteomic analysis of dendritic cells treated with these proteins revealed significant enrichment in immune-related pathways, including interferon and TNF-signaling, as well as metabolic pathways linked to immune activation.

**Conclusions:**

These results demonstrate that three protease inhibitors from bacteria: Ecotin, APRin, and STA function as novel oral mucosal adjuvants capable of modulating immune responses and enhancing antigen immunogenicity.

## Introduction

Vaccination against infectious diseases has significantly reduced mortality and morbidity across the globe (1). Most human pathogens initiate infection at mucosal surfaces; however, licensed vaccines are mainly administered by injection, which preferentially induces systemic immune responses and fails to elicit mucosal immunity (2, 3).

Mucosal immunization can induce both local and systemic adaptive immune responses. Among mucosal routes, oral vaccines are considered ideal because they are needle-free, noninvasive, easy to administer, and associated with higher compliance than injected vaccines. These attributes have the potential to reduce overall costs and enable faster vaccine administration, which are particularly important in resource-limited settings. Despite these benefits, only a few oral vaccines have been licensed for use in humans (4, 5). This is due in part to the restricted features of oral mucosal tissues that make oral vaccine development challenging. Oral mucosal tissues maintain a fine equilibrium and facilitate tolerance induction against environmental and dietary antigens (Ags) while mediating effector responses against pathogens (2, 6). Protection of these surfaces is facilitated by a combination of mechanical, physicochemical, and immunological barriers. Mechanical and physicochemical barriers include the presence of mucus produced by goblet cells, as well as antimicrobial peptides produced by Paneth cells, proteolytic enzymes, and low gastric pH in the gastrointestinal tract (2, 7).

Currently, the eight oral vaccines licensed for human use are either live attenuated (polio, OPV; typhoid, Vivotif^®^; cholera, Vaxchora^®^; rotavirus, Rotarix^®^ and RotaTeq^®^) or whole-cell inactivated formulations (cholera; Dukoral^®^, Shanchol^®^, and Euvichol-Plus^®^) that are less susceptible to intestinal degradation either by replicating in the gut or by virtue of having digestion-resistant bacterial walls. Subunit vaccines are generally safer and less reactogenic than killed or live attenuated vaccines; however, there are no approved human mucosal protein-based vaccines (8, 9). Several issues must be addressed when developing oral subunit protein vaccines, as they generally suffer from high proteolytic digestion and low stability, leading to suboptimal induction of antibody and cellular immune responses (10).

In previous work, we demonstrated that the unlipidated outer membrane protein of 19 kDa (U-Omp19) from *Brucella abortus* can be used as an adjuvant in oral vaccine formulations. U-Omp19 acts as an inhibitor of gastrointestinal proteases, protecting Ags from degradation and increasing the half-life of co-delivered Ags at mucosal inductive sites while recruiting dendritic cells (DCs) and increasing the frequency of mucosal DCs bearing the co-delivered Ag (11–13). Consequently, mucosal Ag-specific immune responses, T helper (Th) 1, and T CD8^+^ responses are enhanced when U-Omp19 is co-delivered orally with different Ags. Likewise, U-Omp19 improves protection against *Toxoplasma gondii* and *Salmonella Typhimurium* challenge when it is co-administered orally with subunit Ags (12, 14, 15). U-Omp19 has also demonstrated adjuvant activity for bacterial and viral Ags delivered by subcutaneous or intramuscular routes (16–18). The protease inhibitor activity of U-Omp19 on lysosomal proteases inside DCs has been linked to its adjuvanticity by the parenteral route (11). These reports demonstrated for the first time the use of a protease inhibitor from bacteria as a vaccine adjuvant. In contrast, viral and parasite-derived protease inhibitors were found to downmodulate immune responses, inducing tolerogenic responses (19). Serine protease inhibitors (serpins) have been identified in parasitic helminths with anti-inflammatory activity (20) and involvement in parasite survival through interference with the host immune response (21).

Based on these previous results, in this work we investigated whether other microbial protease inhibitors, especially bacterial endopeptidase inhibitors, could have immune adjuvant activity. First, *in silico* screening using databases led to the selection of putative protease inhibitors present in human pathogenic bacteria representing different families of protease inhibitors. The selected proteins were then screened for their protease inhibitor activity and immunostimulatory properties. Finally, selected protease inhibitors were studied as oral adjuvants *in vivo* in mice using model and real Ags.

## Results

### *In silico* screening and selection of putative protease inhibitors from bacteria

**Figure 1:**
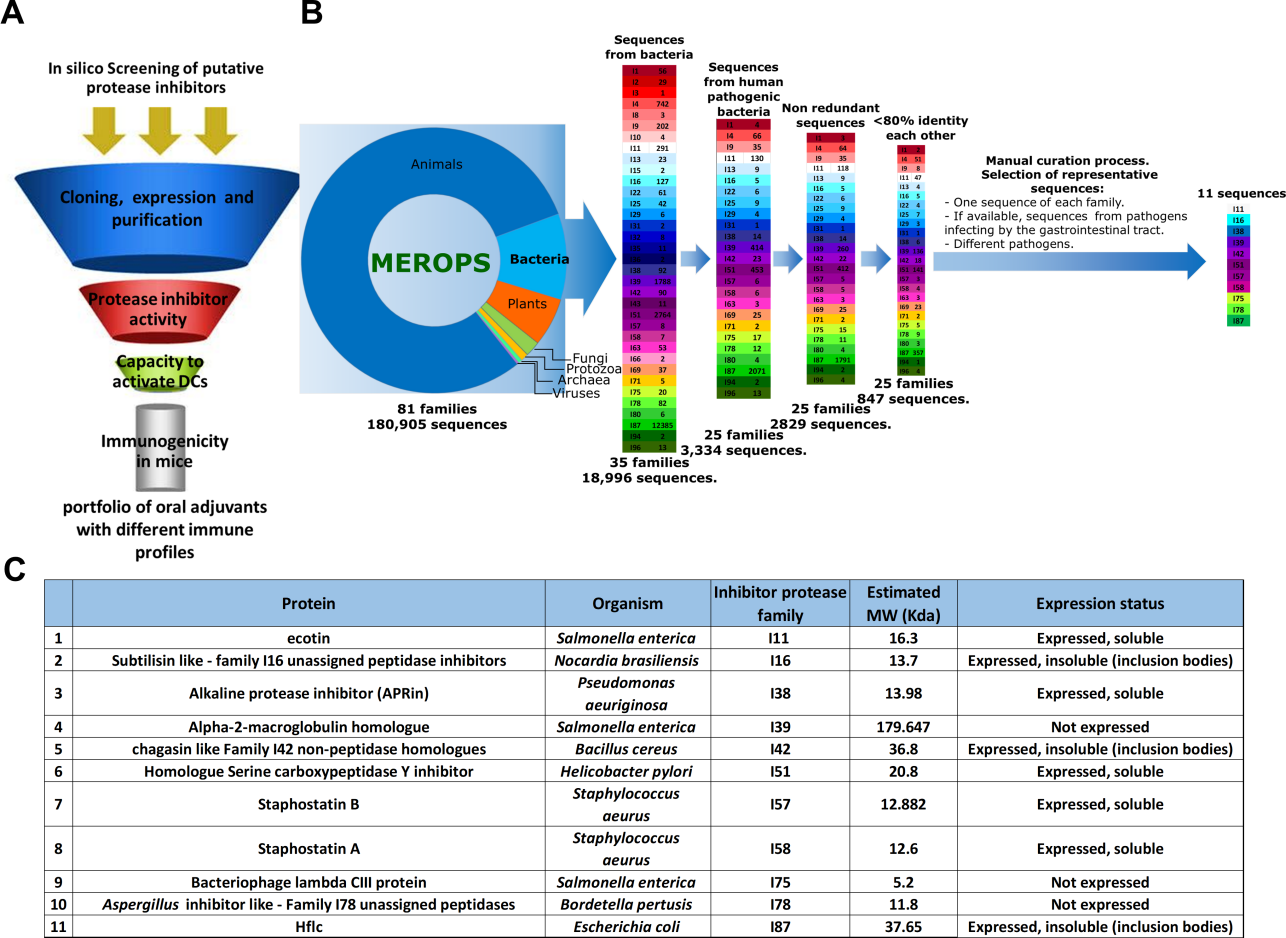
Screening of putative protease inhibitors. **(A)** Schematic representation of the screening. **(B)** Results of the *in silico* screening using the MEROPS database. Eleven sequences were selected to evaluate biological activity and immunostimulatory properties. **(C)** Characteristics of the 11 proteins selected regarding inhibitor family, molecular weight, and expression status.

The strategy for screening and discovery of new compounds is summarized in **Figure 1A**. It began with *in silico* screening to select protein sequences of putative protease inhibitors (PIs) from bacteria. Protease inhibitors can be classified into families based on similarities detectable at the amino acid sequence level (MEROPS database). The *in silico* screening was conducted using the MEROPS database (22) along with literature reports on protease inhibitors. First, sequences were grouped according to their presence in different organisms, and those belonging to bacteria (35 families of PIs) were selected. Then, sequences were grouped according to their presence in human pathogenic bacteria (25 families of PIs). Finally, after removal of redundant sequences, a total of 847 sequences belonging to 25 families were manually curated. Putative protease inhibitors from different PI families and from relevant bacterial pathogens that infect humans were selected (**Figure 1B**). Preferentially, inhibitors from bacteria that enter the body via mucosal surfaces, mainly the gastrointestinal tract, were chosen. Ultimately, 11 sequences of putative protease inhibitors representing different PI families were selected (**Figure 1C**).

Bioinformatic analysis of sequences was performed to evaluate solubility, the presence of signal peptides, and the physicochemical parameters of the recombinant proteins to be expressed in *E. coli*. Of the 11 sequences, five proteins were successfully expressed. Expression was not successful for three of them (alpha-2-macroglobulin, bacteriophage lambda CIII protein, and *Aspergillus* inhibitor-like protein), and the other three were expressed but were found in inclusion bodies (subtilisin-like protein, chagasin-like protein, and HflC; **Figure 1C**). Screening continued with the five soluble, expressed proteins that achieved good purification yields (Ecotin, APRin, staphostatin A, staphostatin B, and serine carboxypeptidase Y inhibitor [ScYi]). Proteins were expressed in *E. coli* and purified by affinity chromatography. The recombinant proteins obtained were run on SDS-PAGE under reducing and nonreducing conditions, showing highly pure protein preparations in all cases (**Supplementary Figure 1**). All recombinant protein preparations were depleted of LPS, and after endotoxin determination, all PI preparations contained <0.1 endotoxin units per mg of protein.

### Selected candidates can inhibit the protease activity of gastrointestinal proteases

**Figure 2:**
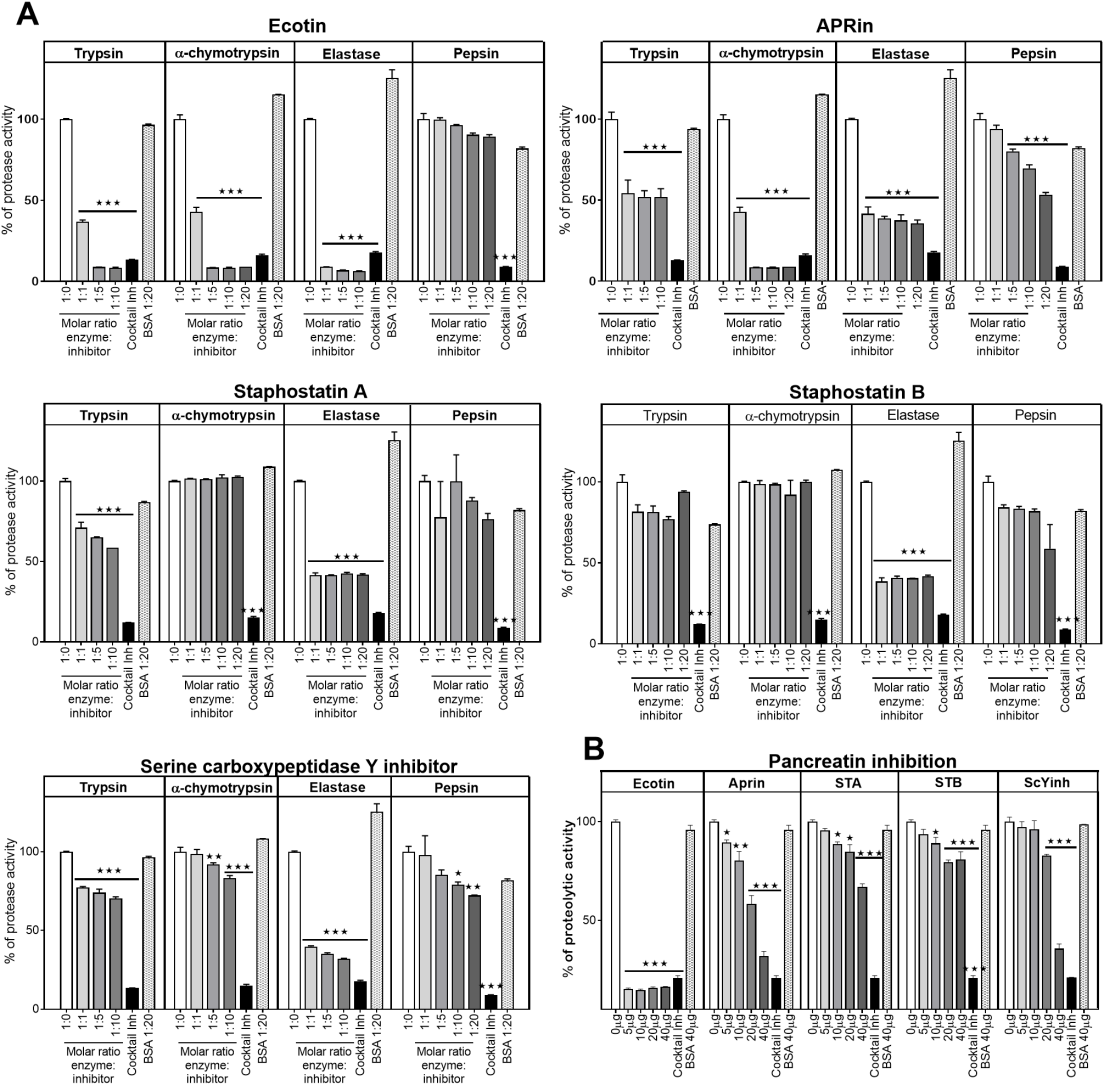
Protease inhibitor activity of proteins. Protease inhibitor activity was determined using the casein–BODIPY-FL assay kit, in which the increase in fluorescence is proportional to proteolytic activity. **(A)** Trypsin, α-chymotrypsin, elastase, and pepsin were preincubated in the optimal buffer for each enzyme for 1 h at different molar ratios of enzyme:inhibitor (1:1, 1:5, 1:10, and 1:20) or without protease inhibitors (1:0). A mammalian protease inhibitor cocktail was used as a positive control and BSA as a negative control at a 1:20 M ratio. **(B)** Pancreatin was incubated for 1h with different amounts of protease inhibitors (0, 5, 10, 20 and 40 µg). A mammalian protease inhibitor cocktail was used as a positive control and BSA (40 µg) as a negative control. The samples were then incubated with 1 µg/mL casein–BODIPY-FL for 1 h. Inhibitor activity is expressed as the percentage of protease activity remaining compared with the 1:0 or 0 µg condition. Bars show mean ± SD of experimental replicates. Lines above the bars include all conditions with equal p values. *p < 0.05; **p < 0.01; ***p < 0.001 vs. 1:0 condition (Kruskal–Wallis test).

The protease inhibitor activity of the five putative PIs was assessed *in vitro*. Activity was evaluated against the four main proteases present in the gastrointestinal tract: trypsin, α-chymotrypsin, elastase, and pepsin. All PIs inhibited at least one of the proteases studied at one or more molar ratios assessed (**Figure 2A**). Ecotin significantly inhibited the activity of trypsin, α-chymotrypsin, and elastase at all molar ratios evaluated but did not inhibit pepsin. APRin exhibited the broadest inhibitory capacity, as all four gastrointestinal proteases tested showed significantly reduced proteolytic activity in its presence, even at low molar ratios. Staphostatin A significantly inhibited trypsin and elastase at all molar ratios assessed but did not inhibit α-chymotrypsin or pepsin. Staphostatin B induced a significant reduction in the proteolytic activity of elastase but could not inhibit trypsin, α-chymotrypsin, or pepsin. ScYi inhibited trypsin and elastase at all molar ratios evaluated but only inhibited α-chymotrypsin and pepsin at higher molar ratios (**Figure 2A**).

We also evaluated whether the protease inhibitors could inhibit a commercial pancreatic extract from pigs (pancreatin). All protease inhibitors were able to reduce the protease activity of pancreatin but with different potencies. Ecotin showed the best performance in inhibiting pancreatin (**Figure 2B**). These results indicate that the five PIs have protease inhibitor activity against gastrointestinal proteases but with different specificities and degrees of inhibition.

### Immunostimulatory capacity of protease inhibitors on dendritic cells *in vitro*

**Figure 3:**
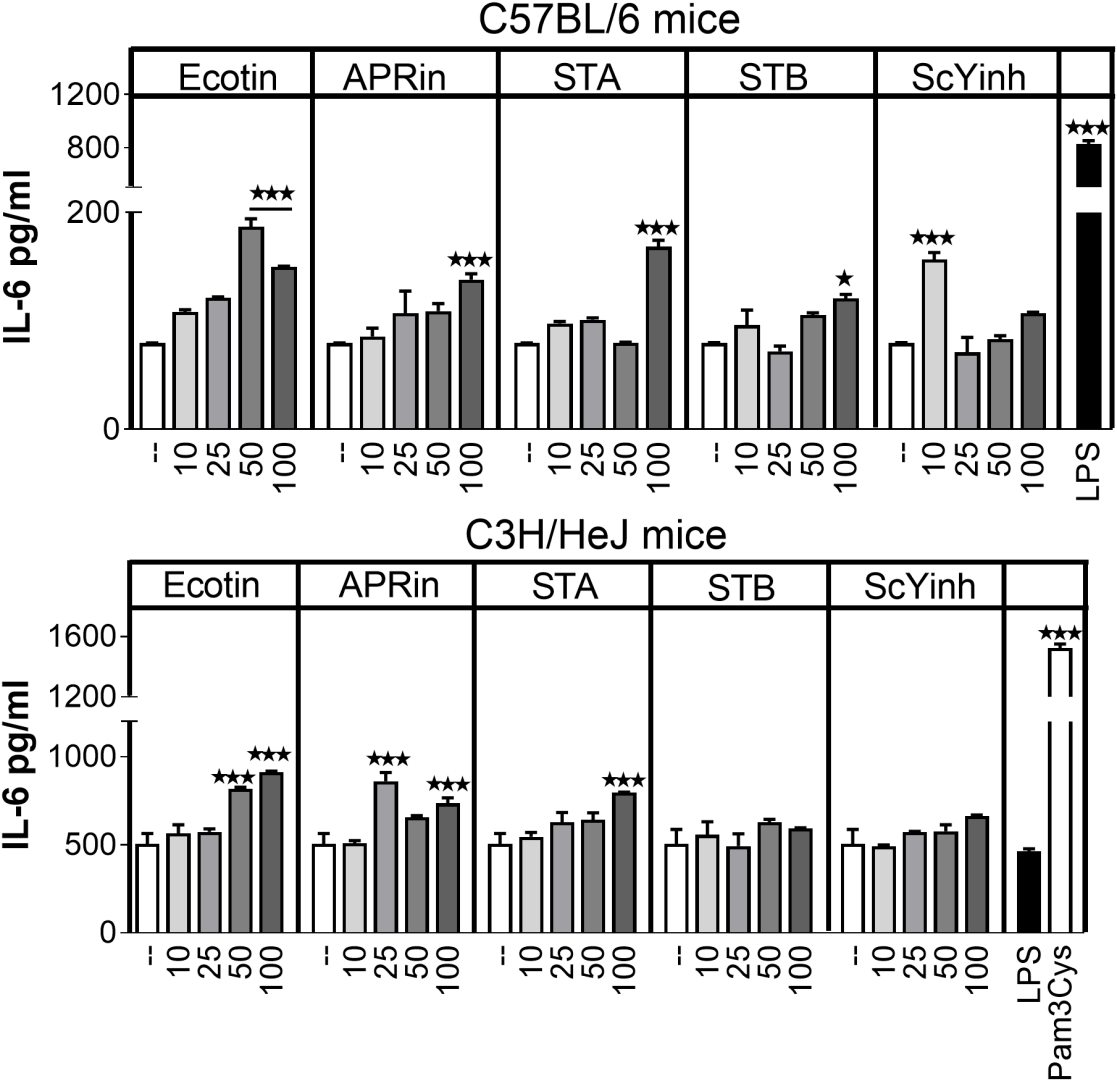
Immunostimulatory properties of protease inhibitors over BMDCs. BMDCs were incubated *in vitro* with different amounts of the selected inhibitors (10–100 µg/mL) for 18 h. IL-6 production was evaluated in the supernatant by ELISA. Stimulation with RPMI was used as a negative control, and stimulation with LPS or Pam3Cys was used as a positive control. Bars show mean ± SEM of experimental replicates. *p < 0.05; ***p < 0.001 vs. 1:0 condition (Kruskal–Wallis test).

Next, the immunostimulatory activity of PIs on bone marrow–derived dendritic cells (BMDCs) from two mouse strains (C57BL/6 and C3H/HeJ) was evaluated. The C3H/HeJ mice have a mutation in the TLR4 gene, which makes them resistant to lipopolysaccharide (LPS) effects. BMDCs were stimulated with different amounts of PIs, and their activation was assessed by measuring IL-6 levels in the culture supernatant using ELISA. All PIs tested were able to activate BMDCs from C57BL/6 mice, but only Ecotin, APRin, and STA were able to activate DCs derived from C3H/HeJ mice (**Figure 3A**). As Ecotin, APRin, and STA were the ones capable of inhibiting gastrointestinal proteases and had immunostimulatory activity independent of TLR4 on DCs, we continued testing them *in vivo*.

### Ecotin, APRin and STA as oral adjuvants increase the immunogenicity of a model Ag *in vivo*

**Figure 4:**
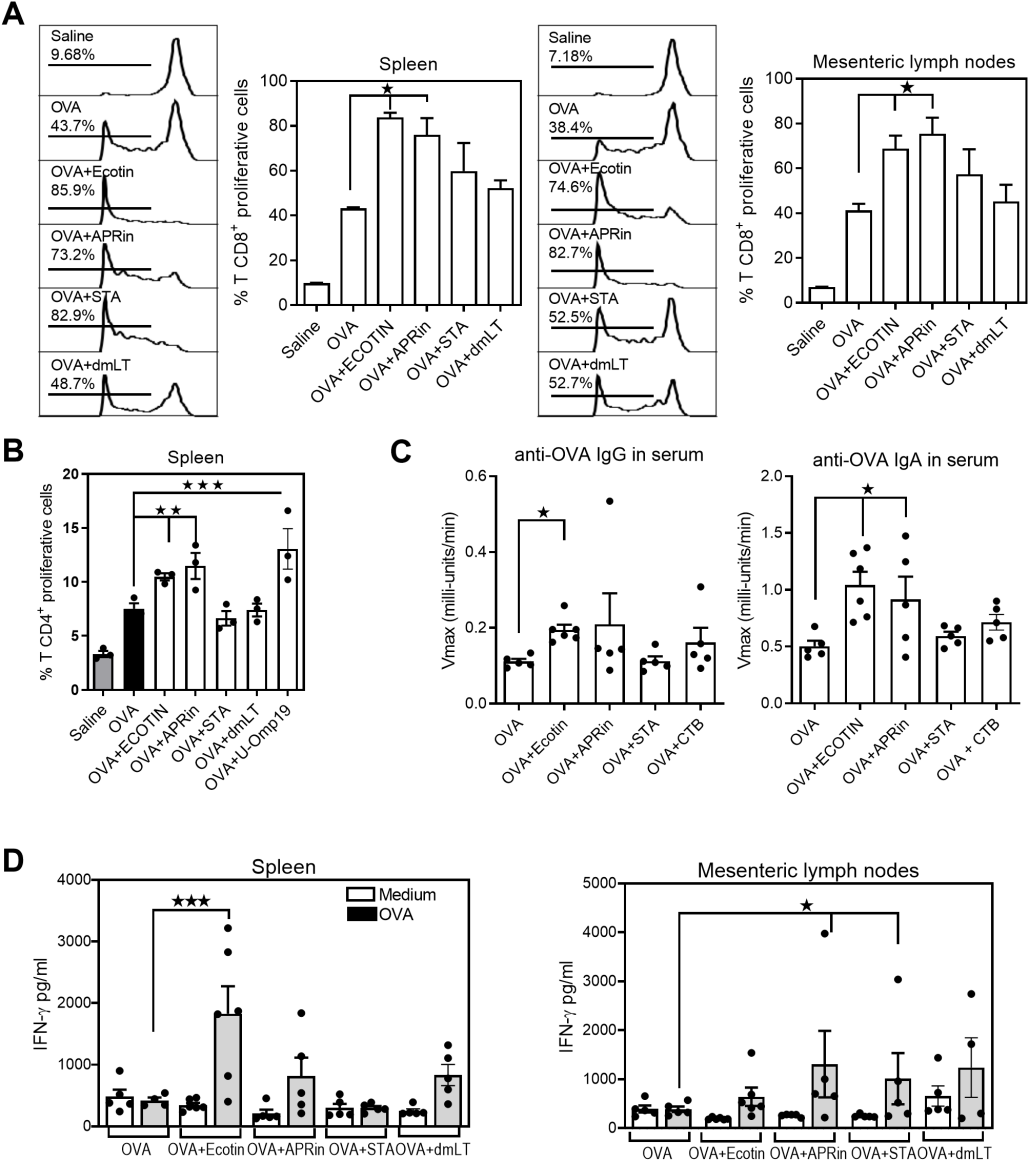
Ecotin and APRin induce T cell proliferation *in vivo* A. **(A)** Proliferation *in vivo* of OT-I CD8^+^ CFSE-labeled T cells after oral administration (*n* = 2–3/group) of saline, OVA, OVA plus Ecotin, OVA plus APRin, OVA plus STA, OVA plus U-Omp19, or OVA plus dmLT. Results are shown as representative histograms, and data are represented as percentage of OT-I CD8^+^ CFSE^+^ proliferative T cells ± SEM. Data are representative of two independent experiments. *p < 0.05 vs. OVA group (one-way ANOVA with Bonferroni multiple-comparison test). **(B)** Proliferation *in vivo* of DO11.10 CD4^+^ CFSE-labeled T cells after oral administration (*n* = 2–3/group) of saline, OVA, OVA plus Ecotin, OVA plus APRin, OVA plus STA, or OVA plus dmLT. Results are shown as representative histograms, and data are represented as percentage of CD4^+^ CFSE^+^ proliferative T cells ± SEM. Data are representative of two independent experiments. **p < 0.01; ***p < 0.001 vs. OVA group (one-way ANOVA with Bonferroni multiple-comparison test). **(C)** Ag-specific IgA and IgG responses induced by protease inhibitors after oral co-administration. BALB/c mice were orally immunized with OVA alone or adjuvants (Ecotin, APRin, STA, and CTB). OVA-specific IgG and IgA were determined in sera 2 weeks after the last immunization. Results are expressed as Vmax (mU/min) ± SEM for each group (n = 5–6/group) (Kruskal–Wallis test). *p < 0.05 vs. OVA alone group. **(D)** Systemic and mucosal T-cell responses induced by oral administration of protease inhibitors as adjuvants. Mice (n = 5–6/group) were orally immunized with OVA alone or plus adjuvants (Ecotin, APRin, STA, and dmLT). Four weeks later, splenocytes and MLN cells from immunized mice were cultured in the presence of complete medium or Ag stimuli for 3 days, and IFN-γ production was analyzed by ELISA. *p < 0.05; ***p < 0.001 vs. OVA group (one-way ANOVA with Bonferroni multiple-comparison test).

The final step of the screening involved evaluating the adjuvant properties of the selected candidates *in vivo* by incorporating them into oral vaccine formulations. First, we studied the ability of the lead candidates to stimulate antigen (Ag)–specific T-cell proliferation *in vivo* when oral co-delivered with chicken ovalbumin (OVA) as a model Ag. Adoptive transfer assays using TCR transgenic OT-I mice were performed to determine *in vivo* the primary clonal expansion of transgenic CFSE^+^-labeled CD8^+^ T cells following oral immunization with OVA alone or with each PI. The experimental double-mutant heat-labile toxin (dmLT) from enterotoxigenic *E. coli* was used as a comparator. After 3 days, mice orally immunized with OVA plus Ecotin or APRin showed greater CD8^+^ T-cell proliferation in the spleen than mice immunized with OVA alone (**Figure 4A**). Interestingly, proliferation in the mesenteric lymph nodes (MLNs) was also higher in groups receiving Ecotin and APRin as adjuvants compared with Ag alone. STA and dmLT did not increase the proliferation of Ag-specific transgenic CD8^+^ T cells compared with OVA delivered alone.

Next, we performed similar experiments using DO11.10 transgenic mice, in which CFSE^+^-labeled CD4^+^ T cells were adoptively transferred to BALB/c mice. The recipient mice were then orally immunized with OVA alone or with PIs. In this case, dmLT and U-Omp19 were used as comparators. Increased CD4^+^ T-cell proliferation was observed in the spleens of mice immunized orally with OVA plus Ecotin, APRin, or U-Omp19 as adjuvants compared with the OVA-alone group (**Figure 4B**). STA and dmLT did not increase the proliferation of Ag-specific transgenic CD4^+^ T cells compared with OVA alone.

In addition, BALB/c mice were immunized orally with OVA plus PIs or cholera toxin subunit B (CTB) as adjuvant on days 0, 14, and 28. OVA-specific antibodies were evaluated in serum two weeks after the last immunization. Mice immunized with OVA + Ecotin showed higher levels of anti-OVA IgG and IgA than the OVA-alone group. Likewise, APRin co-delivered with OVA induced significant levels of anti-OVA IgA in serum. STA and CTB did not increase anti-OVA IgG or IgA in serum (**Figure 4C**).

One month after the last immunization, OVA-specific cellular immune responses were evaluated in the spleen and MLNs after stimulation with the Ag. Mice orally immunized with OVA + Ecotin produced significant levels of IFN-γ in culture supernatants from spleen cells compared with the OVA-alone group. In contrast, in MLN cells, higher production of IFN-γ was observed in groups immunized with OVA plus APRin or STA (**Figure 4D**). These data confirm the potential use of these protease inhibitors as adjuvants to enhance the adaptive immune responses of vaccine formulations delivered orally.

### Ecotin and STA as adjuvants in vaccine formulations containing bacterial Ags increase specific antibody and cellular immune responses *in vivo*

We next evaluated the capacity of the selected PIs to act as adjuvants for real Ags present in licensed human vaccines. The immunogenicity of vaccine formulations containing bacterial Ags—cholera toxin subunit B (CTB) or tetanus toxoid (TT)—and PIs as adjuvants was studied *in vivo* in mice. CTB was used to evaluate antibody (Ab) responses, while TT was used to evaluate cellular immune responses *in vivo.*

**Figure 5:**
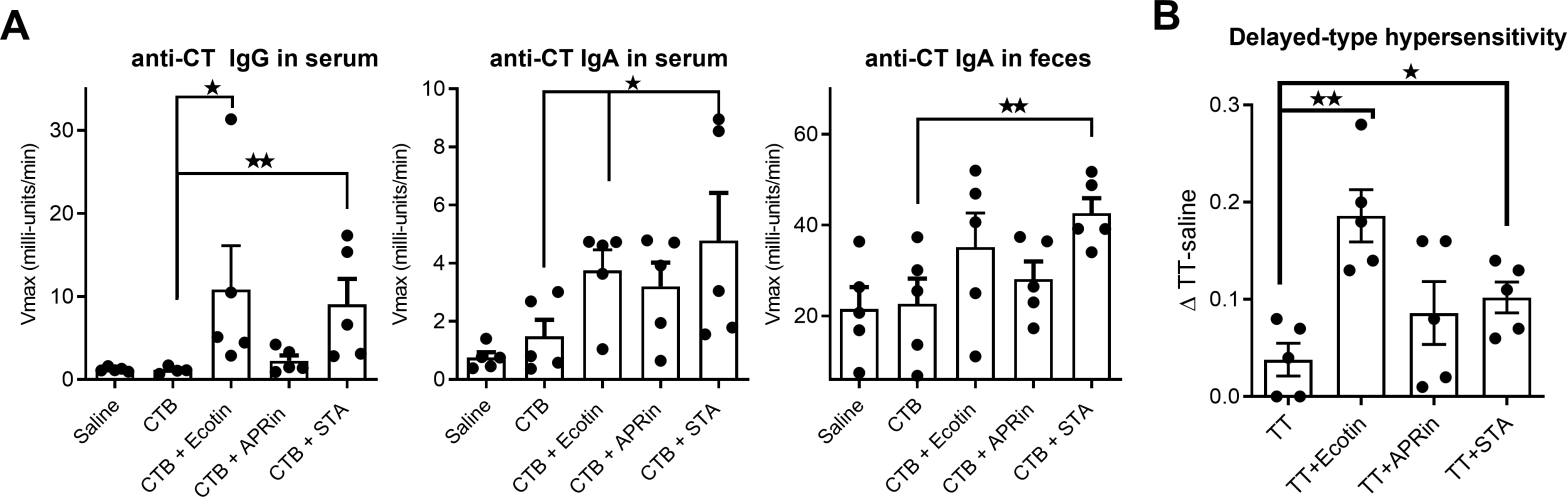
Antibody and cellular responses induced by protease inhibitors after its oral co-administration with vaccine Ags. BALB/c mice (n = 5/group) were orally (intragastrically) immunized with (i) saline, (ii) CT (5 µg) or TT (100 µg) as Ags, or (iii) Ag plus protease inhibitors (150 µg). **(A)** Ag-specific IgG and/or IgA were determined in sera and feces 1 week after the last immunization by indirect ELISA. Results are expressed as Vmax (mU/min) ± SEM for each group (n = 5–6/group) (Kruskal–Wallis test). *p < 0.05; **p < 0.01 vs. Ag alone group. **(B)** In mice immunized with TT as Ag, 3 weeks after the last boost, the delayed-type hypersensitivity (DTH) response was measured by determining footpad swelling 48 h after TT injection into the hind footpad. Data are shown as ΔTT – saline ± SEM for each group. *p < 0.05; **p < 0.01 vs. Ag alone group (one-way ANOVA with Bonferroni post test).

BALB/c mice were immunized orally on days 0, 7, and 14 with CTB alone or in the presence of protease inhibitors, and CT-specific antibodies were evaluated in feces and serum. Mice vaccinated with CTB plus Ecotin or STA showed increased levels of serum IgA and IgG anti-CT antibodies. APRin did not significantly increase serum IgG against CT (**Figure 5A**). Although Ecotin, APRin, and STA increased anti-CT IgA in feces compared with CTB delivered alone, only STA induced a statistically significant increase.

Animals immunized with TT as Ag and co-delivered with Ecotin or STA exhibited an Ag-specific delayed-type hypersensitivity (DTH) response 1 month after the last immunization, while APRin–co-delivered mice did not (**Figure 5B**). DTH is characterized by the recruitment of Ag-specific T cells into tissues, where they are activated by Ag-presenting cells to produce cytokines that mediate local inflammation. These results demonstrate the ability of Ecotin and STA to mediate *in vivo* Ag-specific cellular immune responses following oral co-administration with TT as Ag.

### Ecotin induces the recruitment of different populations of dendritic cells when it is orally co-administered with a model Ag

**Figure 6:**
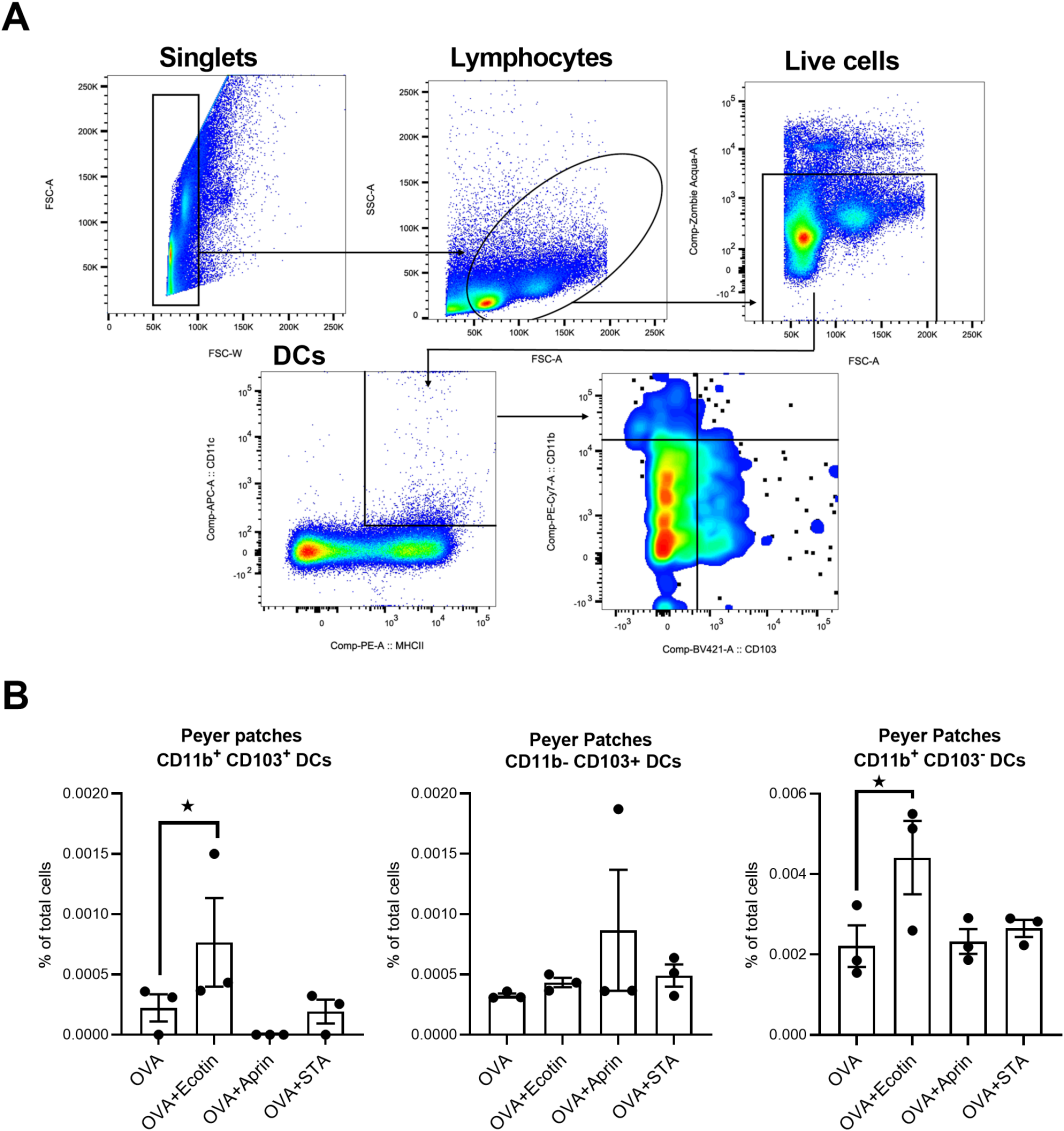
Ecotin promotes dendritic cell recruitment to intestinal inductive sites. BALB/c mice (n = 3/group) were orally (intragastrically) immunized once with (i) saline, (ii) OVA (200 µg), or (iii) Ag plus protease inhibitor (300 µg). Peyer’s patches (PPs) were obtained 6 h later and single-cell suspensions were prepared and stained with fluorochrome-conjugated Abs, including anti-CD11c, anti-MHC-II, anti-CD11b, and anti-CD103, and analyzed by flow cytometry. **(A)** Gating strategy used to analyze data. **(B)** Results are presented as frequency of CD11b^+^ CD103^+^ cells (of total cells) ± SEM. *p < 0.05 vs. OVA alone group (one-way ANOVA with Bonferroni post test).

Since Ecotin, APRin, and STA could induce Ag-specific immune responses in oral vaccine formulations, we next studied the impact of these PIs/adjuvants at mucosal inductive sites at an earlier time point. BALB/c mice were orally immunized with OVA alone or together with Ecotin, APRin, or STA, and 6 h later the Peyer’s patches were collected to evaluate the frequency of different dendritic cell (DC) populations. An increase in the percentage of CD11b^+^CD103^-^ and CD11b^+^CD103^+^ DCs in Peyer’s patches was observed in mice immunized with OVA plus Ecotin (**Figure 6**). This finding demonstrates the capacity of Ecotin to induce the recruitment of DCs to intestinal inductive sites *in vivo*.

### Ecotin, APRin and STA induce changes in the proteomic profile of BMDCs

**Figure 7:**
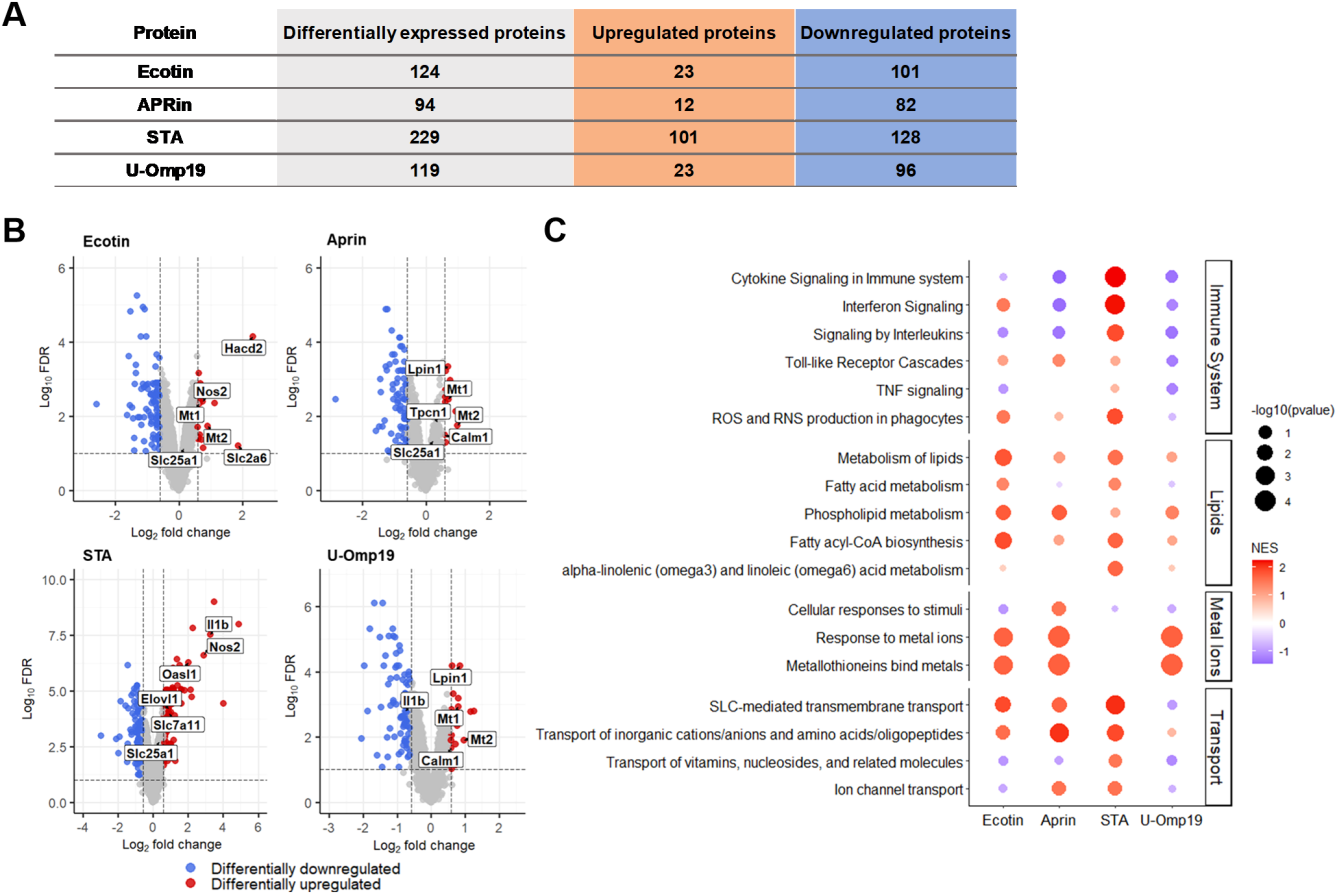
Proteomic profiling of BMDCs stimulated with bacterial protease inhibitors. Bone marrow–derived dendritic cells (BMDCs) were stimulated for 18 h with Ecotin, APRin, STA, or U-Omp19. Proteomic changes were assessed by quantitative LC–MS/MS. **(A)** Number of differentially expressed proteins (DEPs; FDR < 0.1) in each condition, separated into upregulated (orange) and downregulated (blue) proteins. **(B)** Volcano plots showing the distribution of DEPs for each treatment. Significantly upregulated proteins are shown in red, downregulated in blue. Selected immune- and metabolism-related proteins are labeled, including Slc25a1, which was a DEP in all conditions except U-Omp19. **(C)** Gene set enrichment analysis (GSEA) using Reactome pathways, grouped by functional category: Immune System, Lipid Metabolism, Metal Ion Homeostasis, and Transport. Dot size represents the –log10(p-value), and color indicates normalized enrichment score (NES).

Most adjuvants act directly or indirectly on DCs; hence, we analyzed how PIs alter DC protein expression profiles. BMDCs were incubated for 18 h with Ecotin, APRin, STA, or U-Omp19, followed by quantitative LC-MS analysis to identify differentially expressed proteins (DEPs) and affected pathways. A total of 4,461 proteins were identified across all samples. Based on a false discovery rate (FDR) < 0.1, 124 DEPs were found in Ecotin-treated BMDCs, 94 with APRin, 229 with STA, and 119 with U-Omp19 (**Figures 7A, B**).

Gene set enrichment analysis revealed that STA-treated BMDCs showed the strongest enrichment in immune-related pathways, including “Interferon signaling,” “Signaling by interleukins,” and “TNF signaling” (**Figure 7C**). In particular, the “ROS and RNS production in phagocytes” pathway was enriched in BMDCs treated with Ecotin, APRin, and STA, highlighted by the upregulation of inducible nitric oxide synthase (*Nos2*), a key mediator of antimicrobial responses and DC activation. Additionally, all treatments triggered enrichment of lipid-metabolism pathways—such as “Phospholipid metabolism” and “Fatty acyl-CoA biosynthesis”—reflecting metabolic reprogramming associated with DC maturation (**Figure 7C**).

Several solute carrier proteins and ion transport–related proteins, including SLC2A6, CALM1, and SLC7A11, were differentially expressed across all conditions. The mitochondrial tricarboxylate transport protein (SLC25A1), responsible for exporting citrate from mitochondria to the cytosol and serving as a key regulator of lipid biosynthesis and histone acetylation during DC activation, was differentially expressed in BMDCs treated with Ecotin, APRin, and STA (FDR < 0.1) but not with U-Omp19 (**Figures 7B, C**). Although it was not classified as a hit because it did not meet the fold-change criterion established, this consistent trend toward upregulation may indicate potential metabolic reprogramming linked to immunostimulatory responses.

Validation of proteins identified as hits and involved in the main affected metabolic pathways was performed by RT-qPCR (*SLC2A6, HACD2, LPIN1, MT2, NOS2*, and *OASL1*; **Supplementary Figure 2**, **Supplementary Table 1**). Ecotin, APRin, and STA induced both common and distinct proteomic profiles and pathway alterations in DCs, influencing pathways related to maturation and activation under each condition. These findings may explain why all three are able to enhance immune responses, although the nature of these responses is not identical among the inhibitors.

## Discussion

Adjuvants, as critical components of subunit vaccine formulations, are essential to induce immunity and immune memory. There are no approved adjuvants for oral vaccines. Currently, the only subunit Ag incorporated in a licensed mucosal vaccine is cholera toxin subunit B (CTB), included as an additional component of the oral killed whole-cell Vibrio cholerae vaccine Dukoral. Although CTB has in the past been classified as an adjuvant, this definition was complicated by the presence of residual cholera toxin or lipopolysaccharide (LPS) in CTB preparations. Indeed, CTB can mediate immune tolerance to attached or mixed Ags by oral and intranasal routes (8). dmLT is a genetically modified version of the heat-labile enterotoxin (LT) from *Escherichia coli*, designed to retain adjuvant properties while reducing toxicity (23). dmLT has been studied as an adjuvant for oral and intranasal vaccines, particularly against diarrheal diseases like *enterotoxigenic E. coli* (ETEC) and *Shigella* (24, 25). dmLT has shown promising safety and immunogenicity profiles in early-stage clinical trials, particularly for enteric vaccines (26).

Previous work from our group and from others (27, 28) has demonstrated that U-Omp19, a protease inhibitor from *Brucella* spp. with immunostimulatory properties, can function as an oral adjuvant in vaccine formulations (11–13, 16). However, immunostimulatory properties are not a general feature of most studied protease inhibitors. Several protease inhibitors have been introduced for drug delivery to interfere with the degradation of therapeutic peptides and proteins in physiological fluids, as well as their transport across biological barriers (29). Used as drug delivery components, protease inhibitors should not increase the immunogenicity of the co-delivered protein. By contrast, increased immunogenicity, while undesirable in drug delivery, would be advantageous in the context of vaccine development.

Protease inhibitors are grouped according to the catalytic class of protease they inhibit, and following the MEROPS inhibitor classification (22), which classifies them into families based on sequence homology and into clans based on protein tertiary structure. At the time of the screening, there were in MEROPS a total of 180,905 sequences that belonged to 81 families and 40 clans. In our screening, a final step involving manual curation of data was used to select representative sequences for the subsequent experimental screening. *In silico* screening of protease inhibitors from human pathogenic bacteria allowed us to select 11 sequences of putative protease inhibitors from different families belonging to relevant bacteria that enter the body via the mucosa and cause significant diseases in humans.

The most abundant peptidase inhibitors in prokaryotic cells are homologous to alpha-2-macroglobulin (family I39), serine carboxypeptidase inhibitor (family I51), and Ecotin (family I11) (30). Candidates from these three families, among others, were selected in our screening. Most of the protease inhibitors produced by bacteria are either intracellular or periplasmic, but there are some PIs secreted into the medium by certain bacteria. Secreted protease inhibitors are produced either to regulate their own proteases or the proteases of other organisms (31, 32).

Selection criteria based on protein expression and purification yield narrowed the final candidates for functional screening. Five proteins were efficiently expressed with high yield and purity—key factors for incorporating adjuvants into vaccine formulations and enabling large-scale production at low cost. Functional assays confirmed their role as eukaryotic protease inhibitors. Further screening identified three lead candidates with immunostimulatory properties—Ecotin, APRin, and STA—capable of activating dendritic cells independently of TLR4 signaling.

Ecotin from *Salmonella* spp., as a purified recombinant protein, was able to inhibit trypsin, α-chymotrypsin, and elastase. This agrees with protease inhibitors of the Ecotin family that have been described to have wide specificity, being able to inhibit a range of serine proteases with high affinity (33–35). This characteristic may be attributable to the presence of two binding sites in the molecule and structural dimerization of Ecotin (33, 36). Ecotin from enterobacteria and parasites performs a protective role against host digestive proteases and targets host proteases to facilitate infection (31, 37). In this work, we demonstrated that Ecotin was able to recruit CD11b^+^CD103^-^ and CD11b^+^CD103^+^ dendritic cells at Peyer’s patches. It has been reported that both CD11b^+^CD103^-^ and CD11b^+^CD103^+^ contribute to Th1 polarization; in particular, CD11b^+^CD103^+^ DCs have been linked to the induction of intestinal Th17 homeostasis (38). Further studies with larger cohorts will be important to expand these findings.

The alkaline proteinase inhibitor (APRin) from *Pseudomonas aeruginosa* is an inhibitor of the serralysin class of zinc-dependent proteinases secreted by several Gram-negative bacteria (39). This enzyme is capable of degrading a variety of host proteins to enhance the pathogenicity of these organisms (40). We demonstrated that recombinant APRin can inhibit serine proteases from the gut (trypsin, α-chymotrypsin) and pepsin from the stomach. Of note, APRin belongs to the I38 family, which is structurally related to the outer membrane protein of 19 kDa from *Brucella* (Omp19), a broad-spectrum protease inhibitor (41). Staphostatins constitute a family of protease inhibitors reported as highly specific inhibitors of cysteine proteases of *S. aureus* named staphopains (42). This study is the first to describe inhibition of mammalian proteases such as bovine trypsin and elastase by staphostatins A and B. Of all inhibitors tested, the staphostatins showed the lowest inhibitory capacity. Their β-barrel fold may contribute to regulating various proteases, including cysteine, serine, and even metalloproteases (43).

Despite structural and functional differences, Ecotin, APRin, and STA activated dendritic cells and induced Ag-specific immune responses when orally co-administered with model or bacterial Ags. However, each adjuvant elicited distinct immune profiles. Ecotin induced strong CD8^+^ and CD4^+^ T-cell proliferation, systemic and mucosal Th1 responses, and robust IgA and IgG antibody production. APRin shared some CD8^+^ and CD4^+^ T-cell activation properties with Ecotin but mainly stimulated mucosal IgA responses and IFN-γ production in MLNs. STA promoted IFN-γ secretion and both systemic and mucosal IgG and IgA responses, including CTB-specific IgA in feces. Like Ecotin, STA also triggered DTH responses, indicating systemic CD4^+^ T-cell activation. These findings highlight their potential for tailoring immune responses based on specific vaccination needs.

Of note, these protease inhibitors induced distinct proteomic profiles and pathway alterations in DCs that may explain the different immune responses elicited. Proteomic changes observed in DCs following incubation with the inhibitors are closely associated with their functional roles, particularly those observed in metabolic pathways such as glycolysis and lipid metabolism, which are critical for effective immune responses. A key player in this metabolic shift is the glucose transporter GLUT6, encoded by the gene Slc2a6. Upregulation of GLUT6 enhances glucose uptake, promoting glycolysis and subsequent fatty acid biosynthesis, which are critical during inflammatory responses (44). Another significant component is the mitochondrial citrate transporter Slc25a1, which exports citrate to the cytosol in exchange for malate. In the cytosol, citrate is converted into acetyl-CoA, a precursor for fatty acid synthesis, and oxaloacetate, which can contribute to nitric oxide (NO) production via inducible nitric oxide synthase (iNOS) (45). These metabolic pathways are key to DC functions, including lipid biosynthesis for membrane expansion and NO production for antimicrobial activity. Upregulation of GLUT6 and Slc25a1 indicates activation of DCs by the protease inhibitors. These findings underscore the importance of metabolic reprogramming in DC activation and function. Targeting metabolic pathways and associated proteins could offer novel strategies for modulating immune responses, with implications for vaccine development and immunotherapy.

In this work, 11 different putative protease inhibitors from bacteria were selected to be evaluated according to three selection criteria: expression, inhibition, and immunostimulatory properties. Among them, the most promising adjuvant immune responses were elicited by three: (i) Ecotin from *Salmonella*, (ii) APRin from *Pseudomonas aeruginosa*, and (iii) staphostatin A from *Staphylococcus aureus*. Altogether, the findings of this work provide proof of concept that molecules exhibiting both key properties— inhibition of gastrointestinal proteases and immunostimulatory effects on DCs—possess oral immune adjuvant properties and can be selected to induce tailored immune responses.

Although proteases have been proposed as therapeutic targets and studies have postulated the usefulness of mammalian protease inhibitors such as aprotinin for co-administration of oral drugs and prior to oral immunization (46, 47), to our knowledge, except for U-Omp19, there are no other studies of protease inhibitors used as adjuvants in oral vaccine formulations to increase immune responses. Therefore, the results derived from this work, in which three new protease inhibitors from bacteria were found to have oral immune adjuvant properties, add an innovative and original concept to the rational design of oral or mucosal vaccine formulations, opening new possibilities for the use of bacterial protease inhibitors as mucosal adjuvants for vaccines.

## Material and methods

### Ethics statement

All experimental protocols were conducted in agreement with international ethical standards for animal experimentation (Helsinki Declaration and its amendments, Amsterdam Protocol of Welfare and Animal Protection, and the National Institutes of Health [NIH], USA, Guide for the Care and Use of Laboratory Animals). The protocols used were approved by the Institutional Committee for the Care and Use of Experimentation Animals (CICUAE) of the University of San Martín (UNSAM) (Permit Number: 04-2016), Buenos Aires, Argentina.

### Animals

Eight- to twelve-week-old female BALB/c, C57BL/6, or C3H/HeJ mice were obtained from the Animal Facility of the Instituto de Investigaciones Biotecnológicas (IIBio-UNSAM). Mice were housed in appropriate conventional animal care facilities and handled according to the international guidelines required for animal experiments at IIBio-UNSAM. Before and after each intragastric administration or immunization, groups of mice were fasted for 2 h, while water was provided ad libitum and removed only for 2 h following immunization. Oral (intragastric) administrations, intravenous and intradermal injections, and blood collection via the submandibular route were performed by trained personnel using precise and rapid techniques without anesthesia. Animals were euthanized following anesthesia with ketamine (80 mg/kg) and xylazine (16 mg/kg) administered intraperitoneally, followed by cervical dislocation to ensure humane treatment and minimize suffering.

### Antigens and adjuvants

Chicken egg ovalbumin grade V (OVA; Sigma-Aldrich) was used as the model Ag. Recombinant unlipidated (U)-Omp19 was obtained as previously described (48). Lipopolysaccharide (LPS) contamination from U-Omp19 was adsorbed with Sepharose–polymyxin B (Sigma). Endotoxin determination was performed with the Limulus amoebocyte chromogenic assay (Lonza). All U-Omp19 preparations used contained <0.1 endotoxin units per mg of protein. Heat-labile enterotoxin (LT) was provided by John Clements (Tulane University, New Orleans, US). Cholera toxin from *Vibrio cholerae* (CT; Sigma) was reconstituted in water and used as Ag for immunizations. Attenuated double-mutant heat-labile toxin LTR192G/L211A (dmLT) was provided by PATH (Seattle, US) and used as an adjuvant.

### *In silico* screening for protease inhibitors

Protease inhibitors are classified into families based on their evolutionary and structural relationships, incorporated in the MEROPS database (http://merops.sanger.ac.uk/inhibitors/). Using this database and relevant literature, we selected different families of human protease inhibitors present in pathogenic microorganisms representing the various families of protease inhibitors. For families of inhibitors expressed by more than one microorganism, we conducted BLAST analyses to assess homology and selected, for this project, microorganisms whose route of entry is through mucous membranes, mainly the oral route.

The MEROPS database initially contained 180,905 protease sequences from 81 families across various organisms. After selecting bacterial sequences and filtering for redundancy (<80%) and human pathogenicity, a final list of 847 sequences from 25 families of putative protease inhibitors was obtained. From this list, we selected 11 putative protease inhibitors from bacteria: (i) Ecotin from *Salmonella enterica*, (ii) subtilisin-like family I16 unassigned peptidase inhibitors from *Nocardia brasiliensis*, (iii) *Aspergillus* inhibitor–like family I78 unassigned peptidases from *Bordetella pertussis*, (iv) APRin from *Pseudomonas aeruginosa*, (v) chagasin-like family I42 non-peptidase homologs from *Bacillus cereus*, (vi) bacteriophage lambda CIII protein from *Salmonella enterica*, (vii) HflC from *Escherichia coli*, (viii) serine carboxypeptidase Y inhibitor homolog from *Helicobacter pylori*, (ix) staphostatin A from *Staphylococcus aureus*, (x) staphostatin B from *Staphylococcus aureus*, and (xi) alpha-2-macroglobulin homolog from *Salmonella enterica.*

### Expression and purification of the protease inhibitors/adjuvants

Using the nucleotide sequences of the selected putative protease inhibitors, we obtained plasmids encoding the proteins with an N-terminal histidine tag in the pET22+ vector (Novagen, Madison, WI, USA), synthesized by GenScript. These plasmids were transformed into competent BL21 (DE3) cells using the CaCl_2_ method, and transformants were selected on ampicillin-containing media. After induction with IPTG, 9 of the 11 proteins were expressed. In this project, we evaluated only the 5 inhibitors that could be efficiently expressed in soluble form with a high yield and subsequently purified: (i) Ecotin from *Salmonella enterica*, (ii) APRin from *Pseudomonas aeruginosa*, (iii) serine carboxypeptidase Y inhibitor homolog from *Helicobacter pylori*, (iv) staphostatin A from *Staphylococcus aureus*, and (v) staphostatin B from *Staphylococcus aureus.*

LPS contamination from the protease inhibitors was adsorbed with Sepharose–polymyxin B (Sigma). Endotoxin determination was performed with the Limulus amoebocyte chromogenic assay (Lonza). All protease inhibitor preparations used contained <0.1 endotoxin units per mg of protein.

### Determination of protease inhibitor activity *in vitro*

Protease activity was determined using a casein fluorometric kit (EnzChek, Invitrogen). Trypsin (0.965 μM; Sigma), α-chymotrypsin (0.965 μM; Sigma), pancreatic elastase (0.965 μM; Sigma), and pepsin (0.483 μM; Sigma) were incubated with each protease inhibitor at different molar ratios of protease:inhibitor (1:0, 1:1, 1:5, 1:10, and/or 1:20). As a positive control, a mammalian protease inhibitor cocktail (Sigma-Aldrich) was used. Bovine serum albumin (BSA) at a molar ratio of protease:BSA 1:20 was used as a negative control. Each reaction mixture was incubated at room temperature (RT) for 1 h, after which the casein substrate (casein–BODIPY-FL, 1 μg/mL) was added. Fluorescence was measured with a fluorescence plate reader (FilterMax F5, Molecular Devices).

To evaluate whether the protease inhibitors inhibited the proteolytic activity of pancreatic extracts, pancreatin was preincubated with buffer, different amounts of the protease inhibitors, inhibitor cocktail, or BSA as a negative control. The mixtures were then incubated with casein–BODIPY-FL for 1 h or with OVA–DQ (a quenched protein that releases fluorescence upon digestion) for 4 h, and the fluorescence increase was determined.

### Bone marrow-derived DCs stimulation

Dendritic cells were generated from bone marrow (BM) mononuclear cells from wild-type C57BL/6 or C3H/HeJ mice as described (49). To study whether DCs were activated by the protease inhibitors, BMDCs were incubated *in vitro* with different amounts of the selected inhibitors (10–100 μg/mL) for 18 h. IL-6 production was evaluated in the supernatant by ELISA. Stimulation with culture medium (RPMI 1640) was used as a negative control, and stimulation with LPS or Pam3Cys was used as a positive control.

### Adoptive transfer of OT-I or D011.10 cells and *in vivo* CD8^+^ or CD4^+^ T-cell proliferation

Splenocytes from OT-I or DO11.10 mice were labeled with 5 μM CFSE (Molecular Probes) prior to intravenous (i.v.) injection. One day before immunization, 10 × 10^6^ OT-I or DO11.10 cells were injected i.v. into C57BL/6 or BALB/c sex-matched recipients. Transferred mice received a single oral dose of saline, OVA alone (500 μg for BALB/c mice and 1000 μg for C57BL/6 mice), OVA plus plus protease inhibitors Ecotin, APRin, or STA (250 μg), OVA plus dmLT (1 μg); or OVA plus U-Omp19 (150 μg). Five days after immunization, mice were sacrificed, and spleen and mesenteric lymph node cell suspensions were obtained to study proliferation of CD8^+^ or CD4^+^ CFSE^+^ T cells by flow cytometry.

### OVA, CT, and TT immunizations

OVA, CT and tetanus toxoid (TT) immunizations: BALB/c mice (n = 5 per group) were intragastrically (i.g.) immunized with (i) saline, (ii) Ag, or (iii) Ag plus protease inhibitor (150 μg). The Ags used were OVA (100 μg/dose), CT (5 μg/dose), or TT (100 μg/dose). OVA immunizations were on days 0 and 14, and CT immunizations were on days 0 and 28. Prior to i.g. immunization, 200 μL of 10% NaHCO_3_ in water was administered to neutralize stomach pH.

### Determination of antibody levels in serum

Sera were obtained weekly to study CT-specific or OVA-specific antibody responses (IgG and IgA) by indirect ELISA. Ninety-six–well plates were coated with 0.1 μg/well of CT or 1 μg/well of OVA overnight at 4°C. Plates were washed with PBS–Tween 0.05% and blocked with 3% skim milk in PBS for 1 h at 37°C. Plates were then incubated with sera for 1 h (diluted in PBS containing 1% skim milk). Plates were washed and incubated with HRP-conjugated anti-mouse IgA or IgG (Sigma, St. Louis, MO, USA) for 1 h at 37°C. Then, TMB (3,3′,5,5′-tetramethylbenzidine) was added, and absorbance was measured at 450 nm.

### Determination of antibody levels in feces

Feces were obtained weekly to study CT-specific IgA by indirect ELISA. Fecal samples were collected from individual mice using a noninvasive procedure designed to minimize stress. Each mouse was gently handled and placed in a clean, sterile plastic container without bedding or food for a short period (5–15 min). Spontaneous defecation was awaited without applying any physical stimulation or restraint.

Fecal extracts were prepared by suspending eight fecal pellets in 1 mL PBS with 50 µg soybean trypsin inhibitor (Sigma). After homogenization and centrifugation at 4°C, the supernatants of the fecal extracts were supplemented with 1.75 mg BSA, 5 µL PMSF 0.2 M, and 1.5 µL sodium azide 1 M. Samples were stored at −70°C and used for IgA determination by indirect ELISA. Ninety-six–well plates were coated with 0.1 µg/well CT overnight at 4°C. Plates were washed with PBS–Tween 0.05% and blocked with 3% skim milk in PBS for 1 h at 37°C. Plates were then incubated with fecal extracts for 1 h (diluted in PBS containing 1% skim milk). Plates were washed and incubated with HRP-conjugated anti-mouse IgA (Sigma, St. Louis, MO, USA) for 1 h at 37°C. Then, TMB (3,3′,5,5′-tetramethylbenzidine) was added, and absorbance was measured at 450 nm.

### Cytokine production

Spleen and mesenteric lymph node cells from immunized mice (obtained 1 month after the last immunization) were cultured in duplicate in RPMI 1640 (Gibco BRL, Life Technologies, Grand Island, NY) supplemented with 10% fetal calf serum (Invitrogen Life Technologies), 1 mM sodium pyruvate, 2 mM L-glutamine, 100 U/mL penicillin, and 100 µg/mL streptomycin (complete medium), in the presence or absence of stimuli (OVA 20 µg/mL) or complete medium alone. After 72 h of incubation at 37°C in a humidified atmosphere (5% CO_22_ and 95% air), cell-culture supernatants were collected and immediately stored at −80°C until analysis. IFN-γ production was analyzed using mouse ELISA kits according to the manufacturer’s instructions (Pharmingen, San Diego, CA, USA).

### Delayed-type hypersensitivity responses

DTH tests were performed as an *in vivo* index of the elicited cell-mediated immunity. Three weeks after the last i.g. immunization, mice received 30 µg TT intradermally into the left footpad, while an equal volume of vehicle (saline) was injected into the right footpad. After 48 h, the DTH reaction was quantified by measuring the difference between footpad thicknesses using a digital caliper with a precision of 0.01 mm. Animals were handled carefully to minimize stress, and measurements were performed by trained personnel without the use of restraint or causing discomfort. The mean increase in footpad thickness (mm) was calculated as:

left footpad thickness (TT) − right footpad thickness (saline).

### *In vivo* recruitment

BALB/c mice (n = 3/group) were orally (intragastrically) immunized once with (i) saline, (ii) OVA (200 µg), or (iii) Ag plus protease inhibitor (300 µg). Prior to intragastric immunization, 200 µL of 10% NaHCO_3_ in water was administered to neutralize stomach pH. Peyer’s patches (PPs) were obtained 6 h later and single-cell suspensions were prepared. Total viable cells were counted. Cells were stained with a viability dye (Zombie Aqua, BioLegend) and later with fluorochrome-conjugated Abs, including anti-CD11c, anti-MHC-II, anti-CD11b, anti-CD103, or isotype-matched controls, for 30 min at 4°C. Afterward, cells were washed and analyzed by flow cytometry. mAbs were purchased from eBioscience (San Diego, CA), BioLegend (San Diego, CA), and BD Biosciences (Franklin Lakes, NJ).

### Proteomics sample preparation and analysis

BMDCs were generated and used in proteomic experiments only when ≥90% of the population was MHC-II^+^ CD11c^+^ and showed no significant expression of co-stimulatory markers (CD80, CD86). Cells were incubated *in vitro* with 100 µg/mL of the different protease inhibitors for 18 h, while RPMI medium alone served as a negative control. Each condition was performed in independent biological replicates.

Proteins were extracted from the samples, digested with trypsin, and labeled with isobaric stable xisotopes (TMT) to enable multiplexed peptide quantification. The proteomic workflow, including LC–MS/MS acquisition, was carried out at the EMBL Proteomics Core Facility (Heidelberg, Germany). Peptide separation and analysis were performed on a Q Exactive Hybrid Quadrupole-Orbitrap mass spectrometer. Protein quantification was achieved using the IsobarQuant software. Raw data were then analyzed to identify differential expression in each condition.

### Bioinformatics analysis

All bioinformatic analyses were carried out with R (RStudio v. 2024.09.1 + 394). Raw mass spectrometry data were searched against the mouse UniProt protein database to identify each peptide. Raw protein intensity values were filtered and processed, followed by batch effect correction (limma v. 3.54.2) and variance-stabilizing normalization (vsn v. 3.66.0). Differential abundance analysis was performed for each experimental condition relative to the negative control using the empirical Bayes method implemented in limma (v. 3.54.2). Proteins with a false discovery rate (FDR) < 0.1 were considered significant and further analyzed through Reactome pathway enrichment (50) (ReactomePA v. 1.42.0). Data visualization, including volcano plots and pathway representations, was performed with ggplot2 (v. 3.5.1).

### RT-qPCR

RNA was purified from cells using TRIzol (Life Technologies), and cDNA was synthesized to perform quantitative PCR for *SLC2A6, HACD2, LPIN1, MT2, NOS2*, and *OASL1* genes. Briefly, RNA was treated with RQ1 RNase-Free DNase (Promega), and reverse transcription was performed with M-MLV Reverse Transcriptase (Invitrogen). Then, SYBR-based real-time PCR (Applied Biosystems) was performed with forward and reverse primers (Genbiotech). Data were generated using the ΔΔCt method. Relative expression was normalized to that of Actb (β-actin).

### Statistical analysis

Statistical analysis and plotting were performed using GraphPad Prism 9 software (GraphPad Software, San Diego, CA). In experiments with more than two groups, data were analyzed using one-way ANOVA with a Kruskal–Wallis test. A p-value < 0.05 was considered significant. When bars were plotted, results were expressed as means ± SEM for each group.

## Data Availability

The original contributions presented in the study are included in the article/**Supplementary Material**. Further inquiries can be directed to the corresponding authors.
