# Correction: Discovery of protease inhibitors from bacteria as novel adjuvants for oral vaccine formulations

**DOI:** 10.3389/fimmu.2025.1731676

**Published:** 2025-11-03

**Authors:** Lorena M. Coria, Celeste Pueblas Castro, Laura Bruno, Helder I. Nakaya, Karina A. Pasquevich, Juliana Cassataro

**Affiliations:** ^1^ Instituto de Investigaciones Biotecnológicas, Universidad Nacional de San Martín (UNSAM) – Consejo Nacional de Investigaciones Científicas y Técnicas (CONICET), Buenos Aires, Argentina; ^2^ Escuela de Bio y Nanotecnología (EByN), Universidad Nacional de San Martín, Buenos Aires, Argentina; ^3^ Hospital Israelita Albert Einstein, São Paulo, Brazil; ^4^ Institut Pasteur de São Paulo, São Paulo, Brazil; ^5^ Department of Clinical and Toxicological Analyses, School of Pharmaceutical Sciences, University of São Paulo, São Paulo, Brazil

**Keywords:** protease inhibitor, mucosal adjuvant, oral delivery, antigen degradation, mucosal immunity

The title of this article was erroneously given as: “Discovery or protease inhibitors from bacteria as novel adjuvants for oral vaccine formulations”. The correct title of the article is “Discovery of protease inhibitors from bacteria as novel adjuvants for oral vaccine formulations”.

The original version of this article has been updated.

