# Supplementary material for: Discovery of protease inhibitors from bacteria as novel adjuvants for oral vaccine formulations
Source: Front Immunol. 2025 Oct 21;16:1679540. doi: 10.3389/fimmu.2025.1679540 (PMC12620975; doi:10.3389/fimmu.2025.1679540)
Supplement: Supplementary file 1 [file Table1.docx]

**Supplementary material**

**
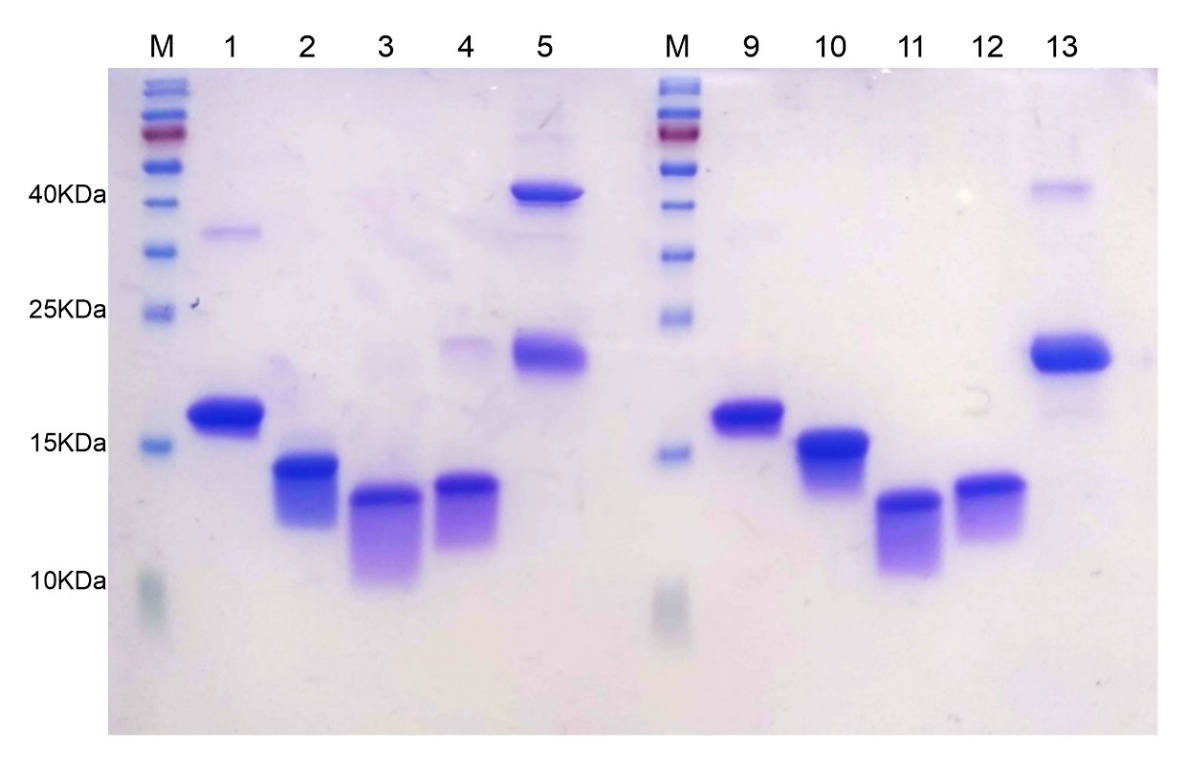
Supplementary Figure 1**

**Supplementary Figure 1.** Reduced and non-reduced SDS-PAGE migration profiles of protease inhibitors samples. M: molecular weight marker. Line 1: non-reduced Ecotin. Line 2: non-reduced APRin, Line 3: non-reduced Staphostatin A, Line 4: non-reduced Staphostatin B, Line 5: non-reduced Y Inhibitor, Line 9: reduced Ecotin. Line 10: reduced APRin, Line 11: reduced Staphostatin A, Line 12: reduced Staphostatin B, Line 13: reduced Y Inhibitor.

**Supplementary Figure 2**


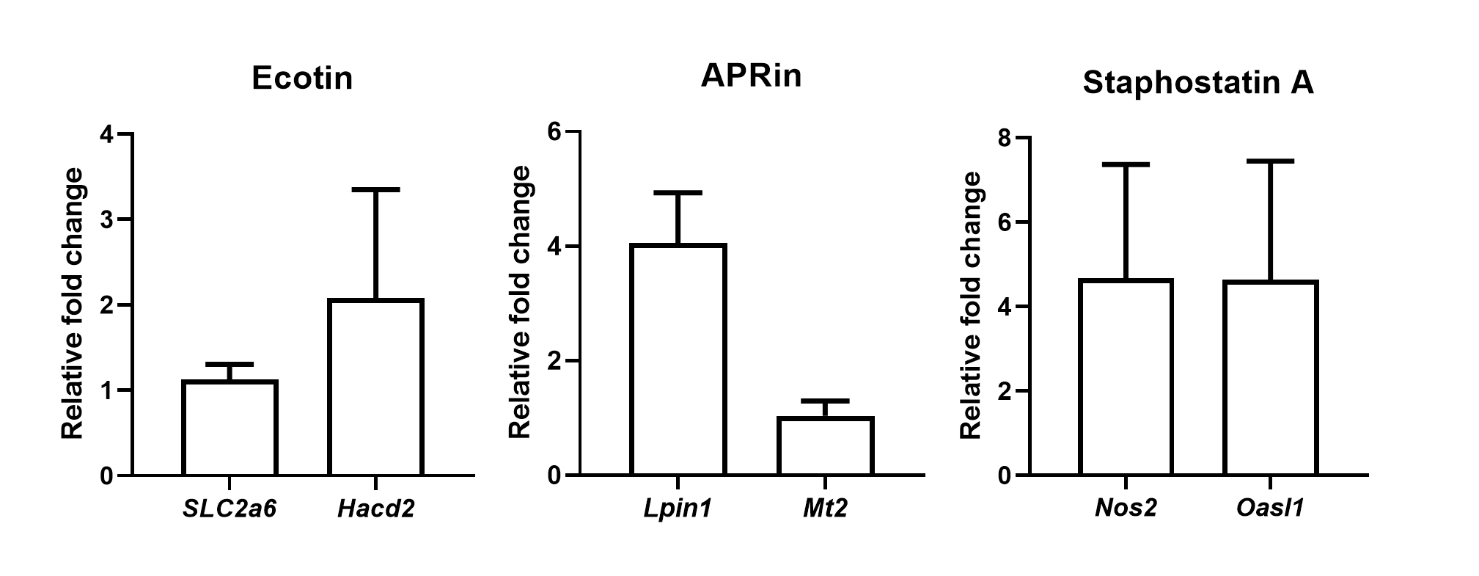


**Supplementary Figure 2.** Gene expression of *SLC2a6, Hacd2, Lpin1, Mt2, Nos2* and *Oasl1* by BMDCs stimulated with Ecotin, APRin or Staphostatin A for 18hs. Data represents fold changes from three biological replicates.

**Supplementary Table 1: Key Proteins with Altered Expression in Proteomic Analysis**
